# On the stoichiometry of zirconium carbide

**DOI:** 10.1038/s41598-020-63037-0

**Published:** 2020-04-14

**Authors:** Claudia Gasparrini, Dhan-sham Rana, Niccolò Le Brun, Denis Horlait, Christos N. Markides, Ian Farnan, William E. Lee

**Affiliations:** 1 Centre for Nuclear Engineering (CNE) & Department of Materials, Imperial College London, South Kensington Campus, London, SW7 2AZ UK; 2 Department of Earth Sciences, University of Cambridge, Downing Street, Cambridge, CB2 3EQ UK; 3 Clean Energy Processes (CEP) Laboratory, Department of Chemical Engineering, Imperial College London, South Kensington Campus, London, SW7 2AZ UK; 4 Université de Bordeaux, CNRS, CENBG-IN2P3, F-33170 Gradignan, France; 5 Nuclear Futures Institute, Bangor University, Bangor, LL57 2DG UK; 6 Present Address: Consorzio RFX, Corso Stati Uniti 4, Padova, 35127 Italy

**Keywords:** Solid-state chemistry, Nuclear fusion and fission, Materials for energy and catalysis

## Abstract

The dependencies of the enhanced thermomechanical properties of zirconium carbide (ZrC_*x*_) with sample purity and stoichiometry are still not understood due to discrepancies in the literature. Multiple researchers have recently reported a linear relation between the carbon to zirconium atomic ratio (C/Zr) and the lattice parameter, in contrast with a more established relationship that suggests that the lattice parameter value attains a maximum value at a C/Zr ~ 0.83. In this study, the relationship between C/Zr atomic ratio and the lattice parameter is critically assessed: it is found that recent studies reporting the thermophysical properties of ZrC_*x*_ have unintentionally produced and characterised samples containing zirconium oxycarbide. To avoid such erroneous characterization of ZrC_*x*_ thermophysical properties in the future, we propose a method for the accurate measurement of the stoichiometry of ZrC_*x*_ using three independent experimental techniques, namely: elemental analysis, thermogravimetric analysis and nuclear magnetic resonance spectroscopy. Although a large scatter in the results (ΔC/Zr = 0.07) from these different techniques was found when used independently, when combining the techniques together consistent values of *x* in ZrC_*x*_ were obtained.

## Introduction

Zirconium carbide (ZrC) is a much-promising material, it has received increased interest recently as an alternative material to silicon carbide (SiC) in nuclear fuel applications^1,2^, in next-generation nuclear fusion reactors^3^, and also as an ultra-high-temperature ceramic to be used in ceramic-metal composite heat exchangers in concentrated solar power (CSP) plants^4,5^.

ZrC (here denoted as ZrC_*x*_) is typically non-stoichiometric as it can contain up to 50% of unoccupied carbon sites^6,7^, it has been found that deviations in the stoichiometry of ZrC_*x*_ can severely affect its thermal and mechanical properties^8,9^. Given its potential in high-temperature applications, it is extremely important to define a method that robustly determines its stoichiometry and purity. The purity of ZrC_*x*_ should always be assessed as the presence of contaminants such as nitrogen or oxygen is detrimental for its performance. For example, if ZrC_*x*_ is to be used as a nuclear fuel coating in a nuclear reactor, any nitrogen contamination should be avoided due to the production of radioactive ^14^C from nitrogen ^14^N inside the reactor^10^.

There are two common methods for defining the stoichiometry of ZrC_*x*_. The first one is to evaluate the C/Zr atomic ratio from the lattice parameter measured by X-ray diffraction (XRD) using the correlation published in Jackson & Lee^6^. The second one is to quantify through the inert-gas fusion technique the carbon content in ZrC_*x*_ and calculate the C/Zr atomic ratio assuming that the sample is free from impurities. Both techniques, however, have limitations and when used in standalone approaches can lead to erroneous stoichiometry estimations, as we will proceed to demonstrate later in this paper.

The need for an established robust method to measure the stoichiometry of ZrC_*x*_ is evident when the relationship between the C/Zr atomic ratio and the lattice parameter is considered. Recently, researchers have published a linear correlation^8,11,12^ between C/Zr atomic ratio and lattice parameter which is in disagreement with the more established relationship from the 1960s–70s showing a maximum at around C/Zr ~ 0.83^13–15^. A significant spread of data exists between ZrC_*x*_ lattice parameter and stoichiometry, which is summarized in the book of Shabalin^16^ and first acknowledged by Mitrokhin *et al*.^15^. The disagreement in the trends reported, linear versus nonlinear, makes it difficult to clearly characterize the stoichiometry of ZrC_*x*_ and therefore link thermophysical properties with ZrC_*x*_. One of the main reasons for the significant spread of data reported can be ascribed to the difficulties in controlling phase contamination due to the solubility of oxygen and nitrogen in the ZrC_*x*_ lattice. Additionally, the presence of oxygen in ZrC_*x*_ lattice gives birth to the oxycarbide class of compounds where there is a direct substitution of carbon (or a carbon vacancy) by oxygen^17–19^. It is also possible for carbon or oxygen to occupy the octahedral interstitial vacancies of the Zr lattice^20,21^. The oxycarbide was shown to exhibit the exact same rock salt structure of ZrC_*x*_ (see the Powder Diffraction File, PDF, 035 0784^22^) with similar^23^ or smaller cell parameters^21^. Gendre *et al*.^17^ observed that oxycarbides with C/Zr ≥ 0.8, have the same XRD pattern as ZrC_*x*_ while oxycarbides with C/Zr ≤ 0.7 could instead display some additional zirconia peaks^17^. If ZrC_*x*_ exists over a wide range of non-stoichiometry (as shown in the phase diagram of by Fernandez-Guillermet^24^ and Jackson & Lee^6^), the oxycarbide, here written as ZrC_*x*_O_*y*_, is also able to exist in the same range of non-stoichiometry. Due to the similarities between the ZrC_*x*_ and the ZrC_*x*_O_*y*_, structures it is challenging to differentiate a pure single phase ZrC_*x*_ sample from an oxygen or nitrogen contaminated sample.

Recent studies^8,11,12,20,25^ have shown a linear relationship or polynomial relationships between lattice parameter and C/Zr atomic ratio and it is remarkable that the authors presenting this new correlations were working on either ZrC_*x*_^8,11,12^ or ZrC_*x*_O_*y*_^20,25^. Zhou *et al*.^26^ discuss the difficulties encountered for the determination of carbon and nitrogen inclusions in ZrC_*x*_ using XRD and they confirmed the expected usefulness of neutron diffraction to determine accurately carbon and oxygen stoichiometries. However, neutron diffraction could not or was not used for the characterization of ZrC_*x*_ specimens in other literature^8,11–14,20,25^, obviously because this technique is quite difficult to access (only ~20 facilities worldwide having an external users program^27^). Other techniques such as Raman spectroscopy that could be used to detect the presence of microcrystalline carbon or amorphous carbon in carbide systems^28,29^, or X-ray photoelectron spectroscopy (XPS) that can also help distinguish the nature of bonds present in the sample, if the metal in the carbide is bonded with carbon, oxygen^30^ or nitrogen^31^, are not often used within the ceramic processing community.

This work presents a critical analysis of the stoichiometry of ZrC_*x*_ as past literature is reassessed depending on the method of sample production, with emphasis on samples manufactured in graphite crucibles or in vacuum and length of heat treatment used. It is found that the linear relationship between the lattice parameter and the ratio C/Zr recently reported on ZrC_*x*_^12^ samples is erroneous as the samples analysed in these studies were ZrC_*x*_O_*y*_ and not ZrC_*x*_ due to a lack of in-depth characterization.

Additionally, the stoichiometries of hot pressed ZrC_*x*_ samples manufactured using commercial powder have been analysed with 3 independent experimental techniques demonstrating that nuclear magnetic resonance (NMR) can detect the presence of graphite when XRD cannot. The comparison of stoichiometry obtained with the three techniques on the same samples elucidates the uncertainty in the stoichiometry definition of ZrC_*x*_ in past and recent literature and provides recommendation for the use of our proposed standardized method for the evaluation of the stoichiometry of ZrC_*x*_ in future studies.

## Experimental Procedure

### Sample preparation and characterization

Hot pressed ZrC_*x*_ samples were manufactured using ZrC commercial powder (3–5 μm, 90% <8 μm with contamination of 0.2% <hafnium <2%, Grade B, H.C. Starck, Karlsruhe, Germany) using the procedure previously described in our previous work^32^ where Set A and B refer to samples hot pressed at 1850 °C and 2000 °C respectively. Set A was made using approximately 42 g of ZrC powder in a 40 mm graphite die, the height was about 0.5 cm. Set B was made using 85 g of ZrC powder filled in the same die, the final height of the disc was 1 cm. The die was lined with graphite foil before powder was inserted to facilitate extraction of the sample. The heating procedure, in argon atmosphere, was set in stages: first heating to 600 °C then a ramp of 20 °C/min was used to reach 1800 °C. A third ramp of 10 °C/min was used to reach the final desired temperature of 1850 °C or 2000 °C. After the desired temperature was reached, samples were hot pressed for 1 h after the 50 MPa of uniaxial pressure was completely applied (it took approximately 15 min). The density was measured with Archimedes method on machined samples cut by electrical discharge machining (EDM) method. To compare the density values obtained with the Archimedes method on different samples tested, the percentage of the theoretical density, TD, was here reported. The TD considered for ZrC was 6.63 g/cm^3^. The density results for Set A and Set B samples are reported in Table 1. Two Set B samples were manufactured using the same powder and same hot pressing procedure and the results from analysis of these two bulk samples are reported.

**Table 1:** Set A and Set B hot pressed samples density results. Measurements were calculated on machined samples cut from bulk discs.

Sample	Sintering temperature (°C)	Density (g/cm^3^)	TD (%)
Set A	1850	6.39 ± 0.01	96.5 ± 0.1
Set B	2000	6.51; 6.58 ± 0.02	97.8; 99.2 ± 0.1

Sample lattice parameters were calculated using XRD characterization with a Bruker D2 Desktop (Massachusetts, USA), with a copper K_α_ source. XRD was performed in the range 2θ: 20–90°. WinPLOTR (Fullprof suite software^33,34^) was used for XRD pattern refinement using Le Bail method and Rietveld refinement. Rietveld refinement cannot be used to obtain information of carbon, oxygen and nitrogen site occupancy due to the fact that XRD data are dominated by the scattering of the cation sublattice, Zr^26^. However, both methods were used to calculate ZrC_*x*_ lattice parameter: the same value was obtained from either Le Bail or Rietveld refinement, nonetheless, Rietveld refinement reduced uncertainties. Sample grain sizes were observed using channelling contrast in a scanning electron microscopy (SEM) using backscattered electron images (BSEI) with a LEO Gemini 1525 FEG-SEM, Zeiss, Germany. Sample surfaces were polished using a Struers MD-Piano cloths 2000 and 4000 grit followed by a 1 µm diamond solution applied on a Struers MD Dac cloth.

### Stoichiometry techniques

The stoichiometry (C/Zr atomic ratio) of samples from Set A and Set B was analysed using three approaches:

1. C/Zr atomic ratio was obtained by fitting the lattice parameter calculated from XRD refinement with the trendline reported in Jackson & Lee^6^ for impurity-free ZrC samples;
2. ZrC_*x*_ stoichiometry was calculated using the full combustion technique in a thermogravimetric analyser coupled with a differential thermal analyser (TGA/DTA);
3. Carbon content in weight % was measured using a carbon analyser and this was subsequently converted into atomic % for definition of C/Zr atomic ratio. NMR was then used to correct C/Zr atomic ratio when inconsistencies were found with results from methods 1 and 2.

### Full combustion method (TGA/DTA)

The full combustion technique using a thermogravimetric analyser coupled with a differential thermal analyser (TGA/DTA) was performed in isotherm mode using a Netzsch STA 449F1 (Netzsch, Germany). A sample from Set A or Set B, previously machined by EDM, was inserted in a TGA/DTA and heated to 1200 °C for Set A and to 1000 °C or 1100 °C for Set B with a rate of 10 °C/min under an argon atmosphere (60 mL/min of argon flow). When the temperature was reached, the sample was kept under argon atmosphere for 20 min to allow the temperature to stabilize before switching to air. The air flow was set to 60 mL/min and the exposure to the oxidizing environment was kept constant for 5 hr or 2 hr and full combustion was reached after approximately 1 hr. The initial mass of the sample, expressed in weight %, here %*m*_ZrC_, and the final mass of the sample, here %*m*_ZrO2_, were recorded. To avoid any possible mass change due to buoyancy affected by gas flow rate insertion, both the argon and air fluxes were set to 60 mL/min. The oxidation of ZrC_*x*_ was considered to follow the reaction:

1 $$ZrC+2{O}_{2}=Zr{O}_{2}+C{O}_{2}$$

where all carbon is converted to CO_2_. From the mass gain evaluated via TGA it was possible to calculate the mass of the final sample by using:

2 $${m}_{{\rm{final}}}=\frac{{m}_{{\rm{ZrC}}}\,\cdot  \% {m}_{{\rm{ZrO}}2}}{ \% {m}_{{\rm{ZrC}}}}$$

where *m*_ZrC_ is the initial sample mass measured before the experiment, %*m*_ZrO2_ and %*m*_ZrC_ are mass percentages (weight %) obtained from TGA analysis. After calculating the final mass of the sample, which is considered to be zirconia, *m*_final_, as all carbon is converted to CO_2_ via Eq. 1, it is possible to derive the mass of zirconium in ZrO_2_ using:

3 $${m}_{{\rm{Zr}}-{\rm{in}}-{{\rm{ZrO}}}_{2}}={m}_{{\rm{Zr}}-{\rm{in}}-{\rm{ZrC}}}=\frac{{m}_{{\rm{final}}}\,\cdot M{W}_{{\rm{Zr}}}}{M{W}_{{{\rm{ZrO}}}_{2}}}$$

where *MW* is the molecular weight of Zr (91.224 g/mol) and of ZrO_2_ (123.218 g/mol). The mass of carbon present in the initial sample is calculated from:

4 $${m}_{{\rm{C}}-{\rm{in}}-{\rm{ZrC}}}={m}_{{\rm{ZrC}}}-{m}_{{\rm{Zr}}-{\rm{in}}-{\rm{ZrC}}}$$

These equations consider that the samples are not contaminated by secondary phases or contaminants. After these calculations, the C/Zr ratio was calculated from:

5 $$\frac{C}{Zr}=\frac{{m}_{{\rm{C}}-{\rm{in}}-{\rm{ZrC}}}/M{W}_{{\rm{C}}}}{{m}_{{\rm{Zr}}-{\rm{in}}-{\rm{ZrC}}}/M{W}_{{\rm{Zr}}}}$$

The uncertainty on the reported value of C/Zr atomic ratio is the standard deviation obtained by performing three calculations using different steady states points for %*m*_ZrC_ and %*m*_ZrO2_.

### Inert gas fusion

Stoichiometry of Set A and Set B was reassessed using an inert gas fusion technique, also called carbon, nitrogen, oxygen analysers using an EMIA 320 V2 and EMGA 820 from Horiba Scientific (Horiba, Japan). This widely used technique allows the quantification of all carbon species present in the ZrC_*x*_ samples but without the possibility of discriminate between bonded carbon (e.g., ZrC) or free carbon (amorphous carbon or graphite). Carbon content in ZrC_*x*_ samples from Set A and Set B was compared to the carbon content present in the commercial powder used. ZrC commercial powder has, according to the manufacturer, a carbon content of 11.6 wt% (11.1% being combined and 0.5% being free carbon). The ZrC commercial powder analysed with the equipment used for this study showed a carbon content of 11.7 ± 0.1 wt%^35^, in line with the manufacturers’ specifications. As described in our previous work^35^ the hot pressed ZrC_*x*_ pellets from Set A had a carbon content of 11.2 ± 0.1 wt%, while pellets from Set B had a carbon content of 11.9 ± 0.1 wt%. The stoichiometry of Set A and Set B from elemental analysis was reported as all other impurities were measured (oxygen and nitrogen) and an accurate (global) C/Zr atomic ratio could be estimated. A small amount of sample, with a mass of approximately 15–70 mg, was used for each measurement. All the uncertainties reported have been calculated as the standard deviation based on 3 measurements.

### Nuclear magnetic resonance (NMR)

Solid-state static ^13^C nuclear magnetic resonance (NMR) spectroscopy was performed on Set B at room temperature to investigate the distribution of carbon environments within the sample and reassess the quantity of carbon bonded in ZrC_*x*_. As ZrC_*x*_ is semi-metallic, shielding from the radiofrequency (RF) waves generated by the NMR can occur if the powder particle diameter is above the skin depth (~33 μm) of the sample. The sample was initially broken using a hammer and further reduction in particle size was achieved using a planetary ball miller (Deco Equipment, China) with stainless steel pots and agate milling balls. The samples were milled until they could be filtered through a 25 µm sieve. The powdered samples were loaded into a 7.5 mm zirconia NMR rotor with aluminium nitride spacers. Static room temperature NMR spectroscopy was undertaken on a Varian Infinity spectrometer at a frequency of 100.603 MHz, for ^13^C NMR in a 9.4 T magnet. The ^13^C NMR spectra were referenced to tetramethylsilane (TMS) via a secondary reference to solid adamantane. Sample spectra were obtained using a Hahn echo pulse sequence, typically around 10000 acquisitions were acquired with a π/2 of 4.70 μs pulse and a pulse delay time of 9 s which were determined to be non-saturating conditions. Peak fitting was undertaken using a Voigt profile, and the Chi squared value was minimized using the Levenberg-Marquardt algorithm in Igor Pro software (WaveMetrics, 10200 SW Nimbus, G-7 Portland, OR 97223, USA). Graphite powder (282863 Sigma-Aldrich, <20 μm, synthetic, purchased from Aldrich Chemistry, Sigma-Aldrich, Merck, Darmstadt, Germany) was used to calibrate the graphite peak observed during analysis of Set B samples, no sample preparation was needed in this case.

## Results

**Figure 1:**
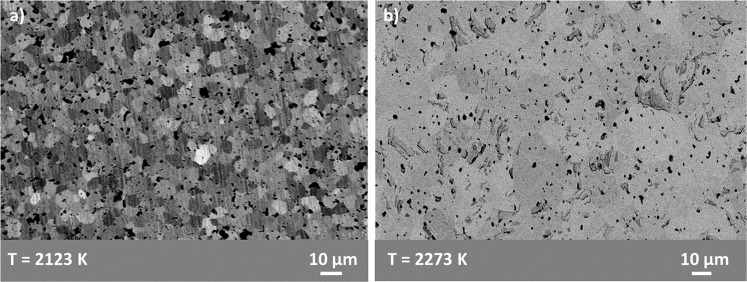
BSEIs of: (**a**) Set A sample showing a TD of 96.5%; and (**b**) and Set B sample showing a TD of 99.2%. Grain microstructure is shown by channelling contrast.

BSEI revealed the grain size and level of porosity of Set A and Set B samples (shown in Fig. 1). The grain size of samples hot-pressed at 1850 °C was smaller than the grain size of samples hot-pressed at 2000 °C. Additionally, samples hot-pressed at 2000 °C (Set B) showed less porosity than samples hot-pressed at 1850 °C (Set A) as can be seen by comparing Fig. 1a,b.

**Figure 2:**
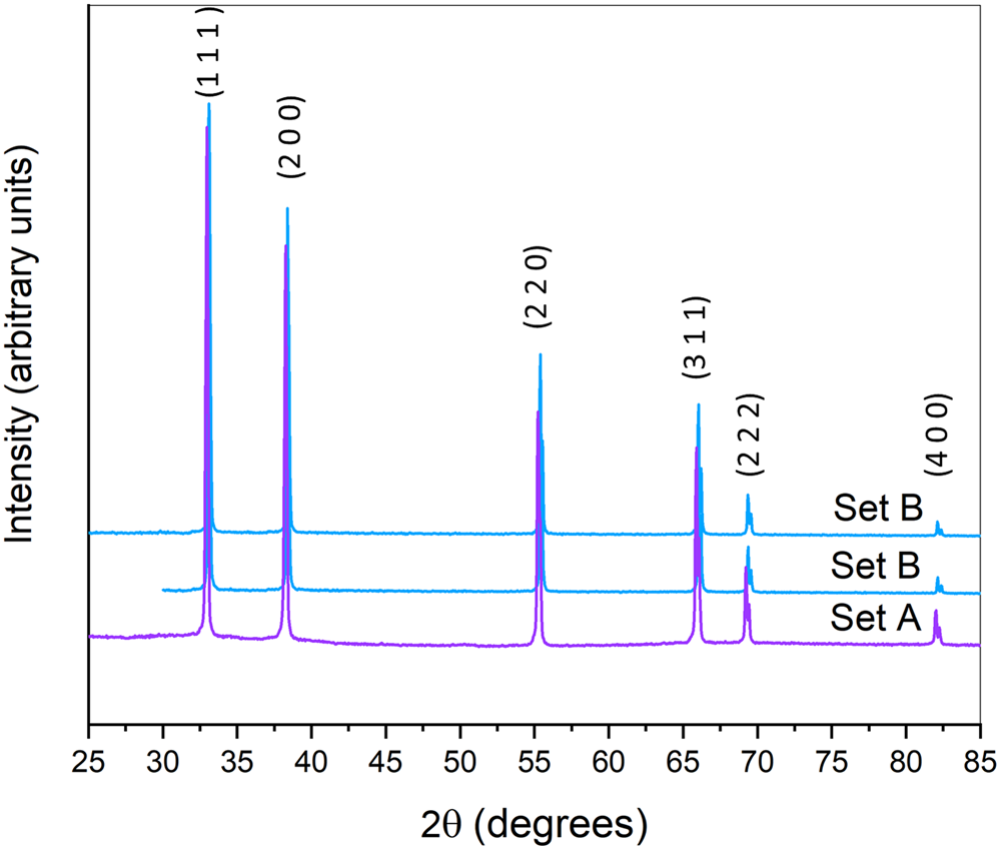
XRD Set A and Set B samples showing ZrC diffraction peaks (PDF 035 0784), no secondary phases were detected.

According to the XRD analysis shown in Fig. 2, in the limit of sensitivity of the XRD apparatus, both Set A and B samples did not display any secondary phase rich in oxygen (such as ZrO_2_) or graphite. Set A showed a carbon content of 11.2 ± 0.1 wt% in line with the combined carbon concentration in the initial ZrC powder. Set B, however, showed a carbon content of 11.9 ± 0.1 wt% indicating the presence of additional free carbon or graphite picked up during the hot pressing stage as commercial ZrC powder only contained 11.6 wt% of bonded and free carbon.

**Figure 3:**
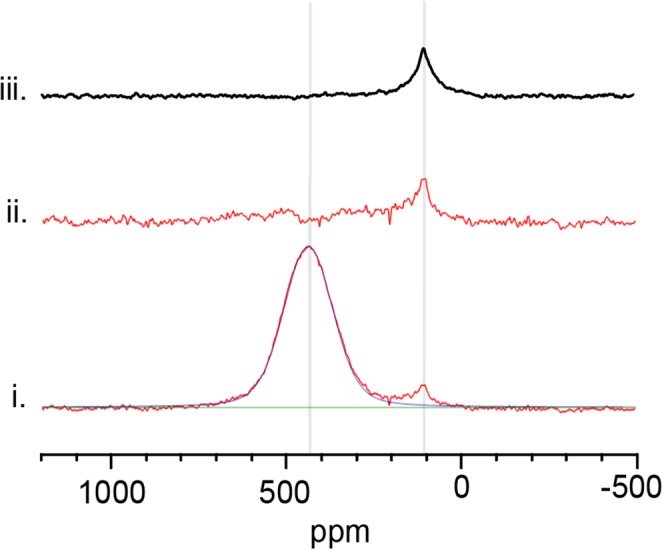
Static ^13^C NMR spectra at room temperature of: (**i**) Set B sample in red, ZrC peak fit (blue) and baseline (green); (**ii**) the residual spectrum post-fitting; and (**iii**) graphite powder. Common peak positions are highlighted with a vertical grey line for comparison.

The nature of the additional presence of carbon measured by inert gas fusion carbon analysis associated with Set B samples could be detected using NMR as this technique can distinguish different carbon local environments. In ^13^C NMR carbon atoms can be directly observed, the integration of a spectral peak corresponds to the proportion of carbon atoms in the associated unique bonding environment and ratio of this with the total spectral signal corresponds to the fraction of carbons present in that environment. NMR was used to analyse the nature of carbon in one Set B sample, the results are shown in Fig. 3.

The static ^13^C NMR spectrum at room temperature in Fig. 3(i) shows that two narrow resonances are observed in Set B sample. The ZrC_*x*_ and graphite resonances are located at ~437 and 113 ppm, respectively. Peak fitting was undertaken on the spectra for the ZrC_*x*_ resonance and it is shown in blue in Fig. 3(i). The post-fit residual spectrum of Fig. 3(i) is shown in Fig. 3(ii). The attribution of graphite to the lower shift resonance, 113 ppm, was confirmed by undertaking static ^13^C NMR of a graphite powder, this is shown in Fig. 3(iii). The separation of the broad line shape of the ZrC_*x*_ and the graphite resonances indicate that graphite present in the sample is entirely disassociated with ZrC_*x*_ structure. The line shape of the ZrC_*x*_ resonance structure, as determined by static ^13^C NMR is homogeneous with no additional sub-resonances. The homogeneity of the ZrC_*x*_ resonance observed in the static spectra is indicative of a single unique carbon chemical environment associated with the ZrC_*x*_ phase. NMR analysis enabled discrimination between carbon in ZrC_*x*_ and carbon disassociated from the ZrC_*x*_ structure which in turn made it possible to recalculate the proportion of carbon atoms in the ZrC_*x*_ sites and hence redetermine the stoichiometry of the sample. By comparing the fitted peak area of the graphite resonance with the total peak area of the NMR spectrum it was possible to correct the C/Zr ratio of ZrC_*x*_ previously calculated by elemental carbon analysis. Static ^13^C NMR on Set A was also undertaken and showed presence of the graphite peak at 113 ppm. Corrected values of stoichiometry for Set A after NMR analysis were also performed and these are discussed in Table 2.

**Table 2:** Summary of hot pressed ZrC samples stoichiometry.

	Elemental analysis C, O and N wt%	C/Zr considering only C wt% (elemental analysis)	C/Zr NMR corrected	C/Zr from TGA	XRD Lattice parameter (Å)	XRD C/Zr (*)
Set A	C = 11.2 ± 0.1 O = 0.95 ± 0.1 N = 0.28 ± 0.1	0.97 ± 0.01	0.96 ± 0.01	0.97 ± 0.01	4.6920 ± 0.0001	0.575 ^(^*^)^
Set B-1	C = 11.9 ± 0.1 O = 0.40 ± 0. 0.03 N = 0.28 ± 0. 0.01	1.02 ± 0.01	0.96 ± 0.01	0.95 ± 0.01	4.6937 ± 0.0004	0.615 ^(^*^)^
Set B-2	C = 11.9 ± 0.1 O = 0.31 ± 0. 0.04 N = 0.26 ± 0.03	1.02 ± 0.01		0.92 ± 0.01	4.6913 ± 0.0003	0.589 ^(^*^)^

C/Zr ratio was measured by TGA method, by elemental analysis (measuring carbon, oxygen and nitrogen in wt %) and by correcting the C/Zr ratio from carbon analysis (wt%) using NMR which enabled discrimination between graphite and bonded carbon. The ratio C/Zr estimated from XRD analysis considered ZrC samples to be free from impurities and by matching the lattice parameter with the trendline in “lattice parameter vs. C/Zr ratio” published in Jackson & Lee^6^ (*).

**Figure 4:**
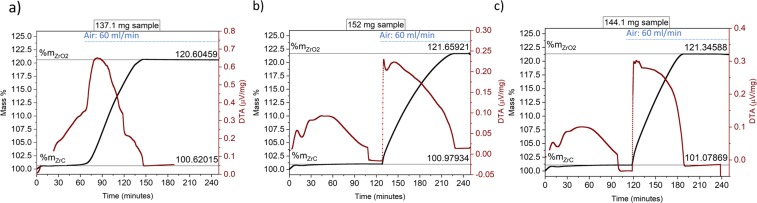
TGA/DTA data obtained during full combustion: (**a**) Set A sample at 1200 °C; (**b**) and (**c**) Set B samples at 1100 °C and 1000 °C, respectively.

TGA/DTA was used to estimate the stoichiometry of Set A and Set B samples using the full combustion method, shown in Fig. 4. The TGA data were used to measure samples stoichiometry by using Eqs. 2–5.

The broad DTA peak in Fig. 4a–c was considered to be related to ZrC oxidation as it coexisted with the TGA curve.

A summary of the stoichiometry results obtained independently from TGA analysis, carbon analysis and NMR analysis are reported in Table 2. The total carbon content of Set B sample, measured by carbon analysis was found to be related to a C/Zr =1.02 ± 0.01, by applying post NMR correction and subtracting the amount of graphite detected in the sample (shown in Fig. 3), the amount of bonded carbon was measured to be related to a stoichiometry of C/Zr = 0.96 ± 0.01 in Set B sample, in agreement with the stoichiometry measured with TGA analysis in Table 2.

Set A stoichiometry value obtained by elemental analysis and TGA was in agreement: C/Zr ratio = 0.97. The mass increase measured by TGA and shown in Fig. 4a indicated a C/Zr value of 0.97 ± 0.01. If oxygen and nitrogen quantities were not considered for the stoichiometry calculation, then C/Zr would have been 0.96 ± 0.01, this indicates the importance of measuring all contaminants when reporting ZrC_*x*_ stoichiometry. When the nitrogen and oxygen elemental analyses on Set A and Set B are considered, these specimens should actually be labelled as an oxynitrocarbides. Only by measuring all contaminants the sample stoichiometry can be determined: Set A, for example has a considerable presence of oxygen and nitrogen and should be labelled ZrC_0.97_O_0.06_N_0.02_.

Another issue occurring when contaminants are not fully quantified is that sample stoichiometry may be erroneously determined using the correlation in Jackson & Lee^6^ for pure ZrC samples. If the correlation in Jackson & Lee^6^ for pure ZrC samples is used on Set A, the stoichiometry is C/Zr = 0.575, a highly substoichiometric sample which is in disagreement with all experimental measurements shown in Table 2. To understand the discrepancy between the C/Zr measured experimentally by TGA and carbon analysis and the fitted C/Zr atomic ratio in Jackson & Lee^6^ from lattice parameter, the relationship between lattice parameter and impurity contents that was first published by Mitrokhin *et al*.^15^. was analysed. The Mitrokhin *et al*.^15^. equation, which was then republished by Jackson & Lee^6^ and Shabalin^16^, is:

6 $${a}_{{{\rm{ZrC}}}_{{\rm{x}}}{({\rm{O}},{\rm{N}})}_{{\rm{y}}}}=4.5621-0.2080{x}^{2}+0.3418x-0.80y(1-x)$$

where *x* corresponds to C/Zr and y is (O + N)/Zr. This equation is only applicable when 0.62 ≤ *x* ≤ 1 and *y* ≤ 0.3, as reported by Mitrokhin *et al*.^15^., therefore it should not be used for C/Zr <0.62 sub-stoichiometric samples.

**Figure 5:**
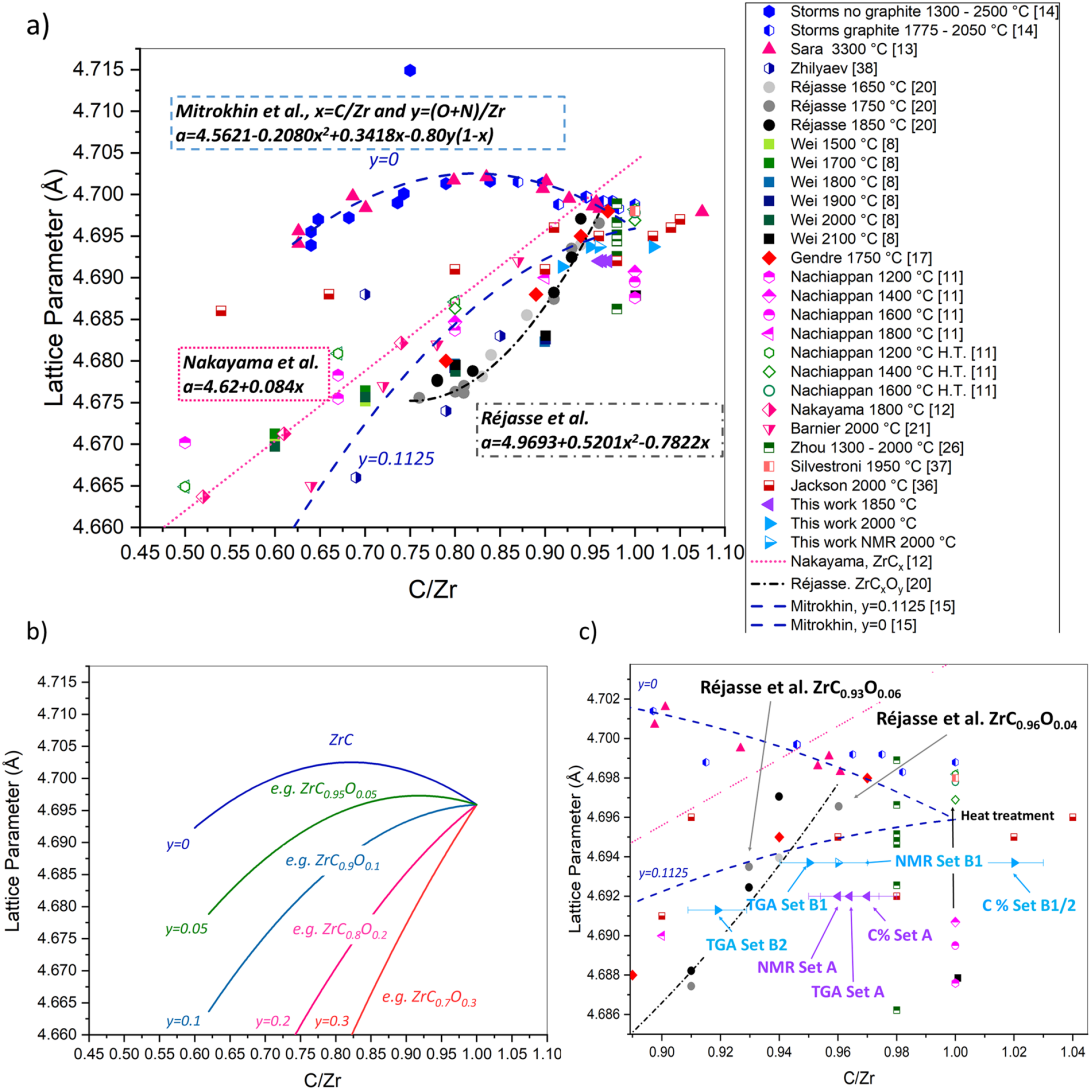
(**a**) Lattice parameter against C/Zr ratio showing Mitrokhin *et al*.^15^ fit, Nakayama *et al*.^12^ fit and Réjasse *et al*.^20^ fit superimposed on zirconium carbide and oxycarbide experimental data, references are in square brackets and H.T. stands for heat treatment. (**b**) Mitrokhin *et al*.^15^ equation plotted over the same range shown in (**a**) with varying oxygen and nitrogen contents: 0 ≤ *y* ≤ 0.3. (**c**) Close-up of the lattice parameter against C/Zr presented in this work shown in (**a**) using three different experimental techniques: TGA, carbon analyser and NMR. Lattice parameter of Nachiappan *et al*.^11^ samples increased after heat treatment as highlighted by the arrow and the “heat treatment” caption.

The stoichiometry of Set A sample, showing a lattice parameter of *a* = 4.6920 Å ± 0.0001, was reanalysed using Eq. 6 considering the boundary condition reported by Mitrokhin *et al*.^15^. and adding the constraint of *x* + *y* = 1 for stoichiometric compounds. The result is that Set A could appear as an oxycarbide with a stoichiometry of ZrC_0.88_O_0.12_ (*x* = 0.88 and *y* = 0.12). This value, ZrC_0.88_O_0.12_, is still in disagreement with the stoichiometry evaluated by TGA and carbon analysis, ZrC_0.97_, see Table 2. The oxycarbide value of ZrC_0.88_O_0.12_ evaluated for Set A using Eq. 6 is, however, not the only possible solution for that given lattice parameter as ZrC_*x*_ allows for non-stoichiometric compounds, therefore ZrC_0.85_O_0.10_ could also be possible. A representation of the quadratic Eq. 6 which covers all possible oxycarbide stoichiometries is highlighted in Fig. 5a,b. The upper limit of the Mitrokhin *et al*.^15^. equation is superimposed upon the experimental data of lattice parameter versus C/Zr from old literature (1960s-1970s)^13,14^, recent literature^8,11,12,17,20,26,36,37^ and the experimental data presented in this work. Figure 5 deliberately combines experimental data from zirconium carbide ZrC_x_^8,11–14,26,36–38^ and oxycarbide ZrC_x_O_y_^17,20,21,38^ studies.

Recent data on oxycarbide production were not reported in Fig. 5 as their stoichiometry was not defined independently but was derived from pre-existing data. Hauser *et al*.^25^. samples stoichiometry, for example, was estimated by fitting their lattice parameter over pre-existing oxycarbide values from Gendre *et al*.^17^, Barnier *et al*.^21^ and Constant *et al*.^39^. The oxycarbide stoichiometry data reported in Gendre *et al*.^17^, Barnier *et al*.^21^ and Constant *et al*.^39^ are in agreement with the equation reported by Mitrokhin *et al*.^15^ for the Zr-C system for *y* ≠ 0.

## Discussion

There is an evident discrepancy in the relationship between lattice parameter and C/Zr ratio in the literature reported from the 1960s and 1970s^13,14^, that shows a peak of the former at a value of C/Zr ~ 0.83^13,14^, and the linear or polynomial correlations found in recent literature^8,11,12,17,20^, which mostly focuses on zirconium carbide ZrC_*x*_ or oxycarbide ZrC_*x*_O_*y*_, production and characterization.

By comparing the experimental method reported in Sara^13^, Storms & Wagner^14^ and Mitrokhin *et al*.^15^, it was noticed that long heat treatments (up to 160 hr) and high temperatures (up to 3300 °C) were used for the production of nonstoichiometric ZrC_*x*_. Storms & Wagner^14^, for example, sintered the powders at 2727 °C before treatment in either vacuum or graphite (at 1325–2050 °C) over long periods (from 1.2 to 160 hr). Mitrokhin *et al*.^15^ reported hot pressing ZrC_0.96_ and ZrH_2_ at 2600 °C for 30 min followed by heat treatment at 2700 °C for 5 hr to obtain non-stoichiometric ZrC_*x*_. Sara^13^ prepared ZrC_*x*_ samples using heat treatments at 2600 °C or 3300 °C for a few hr.

By comparing data reported by Storms & Wagner^14^, shown in Fig. 5a, it became evident that samples that are heat treated in graphite (for 15 hr to 160 hr at temperatures in the range 1775–2000 °C) and samples heat treated in vacuum (for 1.2 to 15 hr at temperatures in the range 1325–2500 °C) can show similar C/Zr versus lattice parameter relationships. The samples heated in graphite may present carbon contamination due to the diffusion of this species in the carbide during heat treatment, especially considering the very long hot-pressing stage in graphite (between 15 hr and 160 hr). Storms & Wagner^14^ did mention that two of the graphite heat treated samples contained free carbon. One key point emerging from the manufacturing process of Storms & Wagner^14^ and Sara^13^ is that very low oxygen contents were reported on the final ZrC_*x*_ samples with stoichiometries in the range 0.648 < *x* < 0.985. The oxygen concentration reported in Storms & Wagner^14^ for ZrC_*x*_ samples ranged between 148 to 1300 ppm which converted in weight% is equal to 0.0148 wt% and 0.13 wt%.

In this work, oxygen impurities reported in ZrC_*x*_ samples from past studies^13,14^ are compared to oxygen impurities reported in recent literature^12,26^ (even though very few authors reported oxygen concentration measurements). It is found that oxygen contamination from current manufacturing routes is much higher than in the 1960s-70s^13,14^. We reassessed the reported stoichiometry of recently manufactured ZrC_*x*_ compounds by taking into account, when published, the oxygen contamination. We found that what was published as ZrC_*x*_ compounds should have been called, instead, ZrC_*x*_O_*y*,_ an oxycarbide. The reassessment of samples stoichiometry from previous studies^12,26^ is shown in Table 3. The samples stoichiometry in Table 3 was calculated converting the reported elemental analysis measured in weight % with atomic % and assuming that no vacancies are present, i.e. *the sum of x and y in ZrC*_*x*_*O*_*y*_ *is equal to 1*. For comparison, samples with similar stoichiometry from Storms & Wagner^14^ are reported to show that Storms & Wagner^14^ produced pure ZrC_*x*_ compounds. Unfortunately, other authors^8,11,40^ recently reporting linear relationship between C/Zr and ZrC_*x*_ lattice parameter have not reported any oxygen and nitrogen analysis on their samples. It is therefore not possible to discern whether their samples were ZrC_*x*_ or ZrC_*x*_O_*y*_N_*z*_. Wei *et al*.^8^ did mention that the discrepancy of their measurements compared to Sara^13^ and Storms & Wagner^14^ could have been induced by presence of impurities such as oxygen but they did not quantify them.

**Table 3:** Corrected stoichiometry from recent studies, the samples when are reassessed to be oxycarbides instead of carbides have been renamed.

Author	Temperature synthesis (°C)	C/Zr reported	Oxygen reported (wt%)	Corrected stoichiometry	Product formed
Zhou *et al*.^26^	2000	0.98	1.011	ZrC_0.94_O_0.07_	oxycarbide
Nakayama *et al*.^12^	1800	0.74	0.67	ZrC_0.74_O_0.04_	oxycarbide
Storms & Wagner^14^	2000 (22.7 hr in graphite)	0.975	0.026	ZrC_0.975_O_0.001_ = ZrC_0.98_	carbide
Storms & Wagner^14^	1800 (3.5 hr in vacuum)	0.736	0.02	ZrC_0.736_O_0.001_ = ZrC_0.74_	carbide

To help the reader discriminate between oxygen content in ZrC_*x*_ and ZrC_*x*_O_*y*,_ compounds, two samples from Storms & Wagner^14^ that were manufactured in the same regime of temperature, one in vacuum and one in graphite, have been analysed using the same method.

Even though Zhou *et al*.^26^ reported that carbon stoichiometry increased and dissolved oxygen decreased with increasing synthesis temperature from 1300 °C to 2000 °C, they reported all their compounds to be ZrC_*x*_ even though they showed a significant level of oxygen even at 2000 °C (see Table 3). From our analysis shown in Table 3 the level of oxygen present in Zhou *et al*.^26^ and Nakayama *et al*.^12^ ZrC_*x*_ samples are of the same order of magnitude of Réjasse *et al*.^20^ oxycarbide samples. We, therefore, renamed Zhou *et al*.^26^ and Nakayama *et al*.^12^ samples oxycarbide compounds, ZrC_*x*_O_*y*_, as shown in Table 3 due to their level of oxygen contamination.

The very long heat treatments and high temperatures used in past manufacturing processes^13–15^ are considered the reason for the high purity ZrC samples synthetized in the 1960s and 1970s^13,14^.

It is worth noting that one of the experimental methods widely used to quantify oxygen content in carbide systems is the inert gas fusion or combustion method technique. In this technique the sample sits in a graphite crucible and is heated to very high temperatures in a helium gas atmosphere to burn all oxygen contaminants into CO/CO_2_. The very long heat treatments in graphite or vacuum at high temperatures conducted by Sara^13^ and Storms & Wagner^14^ may have acted in the same way as the inert gas fusion technique: temperature and time allowed conversion of most of the oxygen in contact with free carbon or graphite to escape the system as CO/CO_2_ leading to oxygen free ZrC_*x*_ samples. These authors^13,14^ both found a relationship between the lattice parameter and ratio C/Zr that exhibited a maximum at ~ 0.83 and this was recently confirmed by Mellan *et al*.^41^. Mellan *et al*.^41^ reported the lattice parameter of ZrC_*x*_ to decrease from *x* = 0.97 to *x* = 1 using computational calculations, as the volume of vacant carbon site (in sub-stoichiometric ZrC_*x*_ compounds) is larger than the corresponding volume of the perfect crystal. This means that the lattice parameter should expand, or increase, for *x* < 1.

The assessment conducted in this work proves that past studies^13–15^ are the only ones that reported production of pure zirconium carbide compounds with very little oxygen impurity, less than 1300 ppm, (we excluded in our discussions one sample reported in Storms & Wagner^14^ that contained 8100 ppm of oxygen with an abnormal lattice parameter of 4.7149, visible as an outlier point in Fig. 5a). The linear relationship found in recent work^8,11,12^ between C/Zr and lattice parameter was derived in studies where the samples instead of being ZrC_*x*_^12,26^ were, unintentionally, oxycarbides: ZrC_*x*_O_y_, as seen in Table 3. The presence, or contamination, of oxygen in ZrC_*x*_ samples produced using commercial ZrC powder like in Nakayama *et al*.^12^ work and this work (see, for example, Set A oxygen and nitrogen analysis in Table 2 relating to ZrC_0.97_O_0.06_N_0.02_) or by mixing ZrH_2_ and carbon black^26^ could be related to the manufacturing process used nowadays, which is shorter (less than 5 hr^8,11,26^ or even only a few min^12,36^) and conducted at lower temperatures (≤ 2100 °C) than the manufacturing process used in the past (up to 160 hr^14^ and 3300 °C^13^). The combination of short reaction and sintering time combined with low temperatures have induced production of contaminated ZrC_*x*_ samples hosting oxygen impurities, therefore being in reality oxycarbides.

Before considering changes in mechanical and physical properties of ZrC_*x*_ with stoichiometry, samples should be correctly characterized, and the stoichiometry be accurately reported considering oxygen and nitrogen contamination. This will help reducing the large scatter of data present to date on thermophysical properties of ZrC_*x*_.

Our study showed that the trend reported by Mitrokhin *et al*.^15^ and republished in Jackson & Lee^6^ for pure ZrC, is no longer able to represent the experimental data reported in recent studies^8,11,12^ (see Fig. 5a). The reason was not related to a novel linear relationship of the lattice parameter with C/Zr atomic ratio recently reported but, instead, was due to the erroneous labelling of samples stoichiometry: recent literature who reported ZrC_*x*_ have unintentionally produced oxycarbides^12,26^ instead of carbides (see Table 3).

The increase of lattice parameter of ZrC_*x*_ samples after post heat treatment reported by Nachiappan *et al*.^11^ agrees well with our hypothesis that ZrC_*x*_ samples manufactured nowadays contain contaminants. It is well known that the lattice parameter of oxycarbides increases with the progressive removal of oxygen^21^, therefore, by heat treating samples in Nachiappan *et al*.^11^ study, most of the oxygen contaminants could leave the system as CO/CO_2_. In Fig. 5c it was highlighted the change in lattice parameter of heat treated samples from Nachiappan *et al*.^11^ that did not quantify the presence of contaminants in their samples. After the heat treatment, their samples presented similar lattice parameter to stoichiometric samples from Storms & Wagner^14^ which indicated a purification process during heat treatment.

In this work we have used three experimental techniques to measure the stoichiometry of commercial ZrC_*x*_ hot pressed samples, which contain oxygen and nitrogen contaminations, in an effort to find the best method: TGA, inert gas fusion and NMR. TGA and carbon analysis C/Zr atomic ratio were in agreement on Set A samples even though TGA was unable to measure small levels of impurities such as free carbon and oxygen. Set B samples presented significant non bonded carbon as C/Zr = 1.02 ± 0.01 measured by inert gas fusion analysis and the stoichiometry measured by TGA giving C/Zr = 0.95 ± 0.01 disagreed. NMR was used in this case to quantify the amount of graphite in the sample and to correct the C/Zr ratio. The NMR corrected value of C/Zr = 0.96 ± 0.01 was in agreement with the TGA method for both Set A and set B samples.

**Figure 6:**
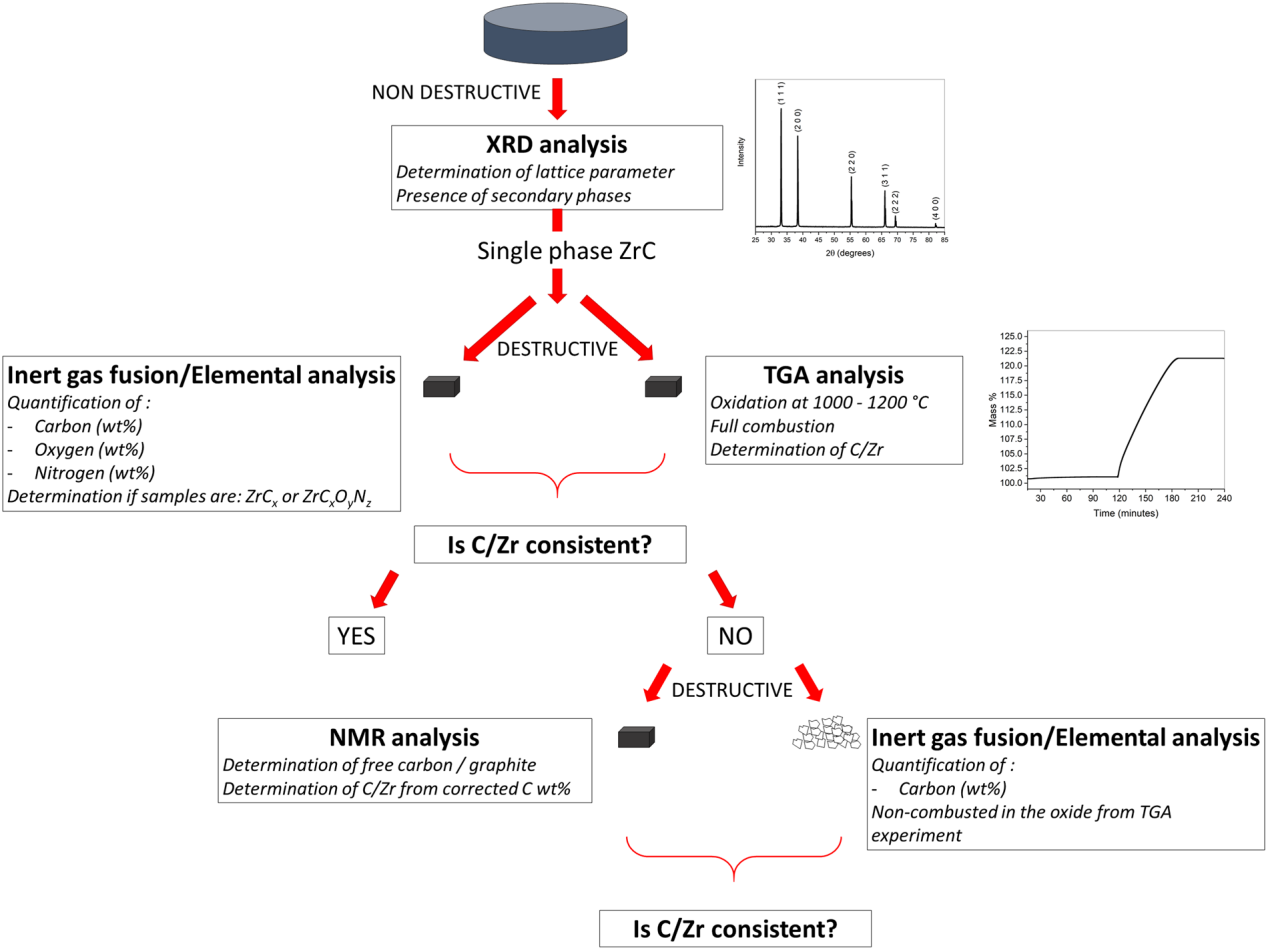
Proposed method to assess ZrC stoichiometry.

We have found that to have a clear understanding of sample stoichiometry in ZrC_*x*_ systems multiple techniques should be used together, and the relationship between C/Zr atomic ratio and lattice parameter published by Mitrokhin *et al*.^15^ and Jackson & Lee^6^ should be used only if the concentration of carbon, oxygen or nitrogen is known. We propose a method to address stoichiometry of ZrC_*x*_ in light of the results from this work and the review on past and recent literature, this is shown in Fig. 6.

In order to evaluate the stoichiometry of a ZrC_*x*_ sample correctly, we propose the following steps:

1. An elemental carbon, oxygen and nitrogen analysis needs to be conducted and the weight % must be converted to an atomic % to determine whether carbides or oxynitrocarbides are present.
2. TGA can be used as a reliable method to determine the stoichiometry of carbides if the sample is mostly single phase (ZrC_*x*_) or if secondary phases are known (e.g., composition of ZrO_2_).
3. NMR needs to be conducted when discrepancies arise between elemental analysis and TGA analysis. NMR allows correction of stoichiometry from elemental analysis as free carbon or graphite can be identified and quantified.
4. Finally, a comparison between the lattice parameter value obtained by XRD and the relationship published by Mitrokhin *et al*.^15^ equation should be performed to validate this theory.

Additionally, we suggest that long heat treatments at high temperature should be used to manufacture pure ZrC_*x*_ samples as the temperature and time seem to be keys in reducing any remaining oxygen contaminants. Lastly, care should be used when storing and handling carbides powders in air during sample preparation as oxygen contamination is difficult to avoid.

## Conclusions

This paper critically analysed the relationship between the atomic ratio C/Zr and the lattice parameter of ZrC_*x*_ samples. It was found that recent literature reporting on a new linear relationship between these two parameters erroneously did not consider contamination of oxygen and nitrogen in the ZrC_*x*_ samples under investigation. We reassessed, where possible (i.e., when the quantification of contaminants was reported), the stoichiometry of ZrC_*x*_ samples that reported a linear relationship between the ratio C/Zr and the lattice parameter and found that those samples were actually ZrC_*x*_O_*y*_. Additionally, it was noticed that very little oxygen contamination was found in older literature concerning ZrC_*x*_ due to the specific manufacturing routes that consisted of very long heat treatments (up to 160 hr) at high temperatures (up to 3300 °C), unlike the manufacturing methods used more recently.

The relationship of the atomic ratio C/Zr and the lattice parameter is not trivial and it is not possible yet to define a single and universal technique which is able to characterize the stoichiometry of ZrC_*x*_ and ZrC_*x*_O_*y*_N_*z*_ in one single stage. In our study, we used three independent experimental techniques to identify consistent values for ZrC_*x*_ stoichiometry. If each method was used alone, instead, a scatter on the atomic ratio as high as ΔC/Zr = 0.07 were found. From this work, we established that a chemical quantification of the carbon, oxygen and nitrogen contents should always be conducted, and the results should be compared with the TGA method which was found reliable in confirming the stoichiometry obtained by elemental analysis. In the case of a discrepancy between TGA and elemental analysis, NMR should be used to correct the final stoichiometry, and the oxide from TGA analysis should be analysed to confirm presence of non-combusted carbon species (as shown in the Supplementary Information).

It is clear that a standardized procedure should be established in order to allow reliable comparisons between stoichiometry and the mechanical and thermophysical properties of ZrC_*x*_. We hope that our proposed method will help future researchers to fully and accurately characterize sample stoichiometries and link these data to other physical or chemical material properties.

## Supplementary information

Supplementary information.

## Data Availability

The datasets generated during and/or analysed during the current study are available from the corresponding author on reasonable request.
