# Supplementary material for: On the stoichiometry of zirconium carbide
Source: Sci Rep. 2020 Apr 14;10:6347. doi: 10.1038/s41598-020-63037-0 (PMC7156448; doi:10.1038/s41598-020-63037-0)
Supplement: Supplementary file 1 — Supplementary information. [file 41598_2020_63037_MOESM1_ESM.pdf]

On the stoichiometry of zirconium carbide

Claudia Gasparrini[a], Dhan-sham Rana[b], Niccolò Le Brun[c], Denis Horlait[d], Christos N. Markides[c], Ian Farnan[b] and William E. Lee[a,e]

Corresponding author: c.gasparrini14@imperial.ac.uk

[a] Centre for Nuclear Engineering (CNE) & Department of Materials, Imperial College London, South Kensington Campus, London SW7 2AZ, U.K.

[b] Department of Earth Sciences, University of Cambridge, Downing Street, Cambridge CB2 3EQ, U.K.

[c] Clean Energy Processes (CEP) Laboratory, Department of Chemical Engineering, Imperial College London, South Kensington Campus, London SW7 2AZ, U.K.

[d] Université de Bordeaux, CNRS, CENBG-IN2P3, F-33170 Gradignan, France

[e] Nuclear Futures Institute, Bangor University, Bangor LL57 2DG, U.K.

Supplementary information

The disagreement between C/Zr ratio calculated from TGA and elemental analysis for Set B samples was further investigated, given that TGA method was able to characterise correctly samples stoichiometry (its result was in agreement with NMR analysis, see Table 2).

The hypothesis used to calculate ZrC_x stoichiometry with the TGA method was that the initial sample presented no excess carbon or impurities, see equation 1. It was reported, however, that Set B presented an excess of carbon as measured by elemental analysis (11.9 wt% against 11.6 wt% of bonded and free carbon reported by the powder manufacturer). The presence of non combusted carbon, in concentration of 0.50 ± 0.02 wt%, was measured by elemental analysis on the oxide produced at the end of the TGA/DTA test shown in Figure 4. This excess carbon cannot be unreacted ZrC_x as the combustion reaction in Figure 4 was completed before the end of the test: both mass gain and change in temperature stabilised. The non combusted carbon measured on the pulverized ZrO_2 powder could be related to the free carbon present in the initial powder (in quantity 0.50 wt% as stated by Starck) or to the graphite phase observed by NMR analysis. Given the consistency in stoichiometry calculated between NMR and TGA it is deduced that the secondary phase of carbon present in the sample did not oxidise during the TGA test. In this way sample stoichiometry could be measured correctly as the free carbon/graphite contribution was not considered in the calculation shown in equations 1-5. The fact that the TGA test performed in air at 1000 °C was not able to combust all carbon species present in the sample should be further investigated as it may be that ZrC_x samples with different content of free carbon or graphite will behave differently.
